# Anisotropic Radiation in Heterostructured “Emitter in a Cavity” Nanowire

**DOI:** 10.3390/nano12020241

**Published:** 2022-01-13

**Authors:** Alexey Kuznetsov, Prithu Roy, Valeriy M. Kondratev, Vladimir V. Fedorov, Konstantin P. Kotlyar, Rodion R. Reznik, Alexander A. Vorobyev, Ivan S. Mukhin, George E. Cirlin, Alexey D. Bolshakov

**Affiliations:** 1 Center for Nanotechnologies, Alferov University, Khlopina 8/3, 194021 Saint Petersburg, Russia; alkuznetsov1998@gmail.com (A.K.); kvm_96@mail.ru (V.M.K.); burunduk.uk@gmail.com (V.V.F.); konstantin21kt@gmail.com (K.P.K.); moment92@mail.ru (R.R.R.); alex.spbau@mail.ru (A.A.V.); imukhin@yandex.ru (I.S.M.); george.cirlin@mail.ru (G.E.C.); 2 Department of Physics, ITMO University, Kronverkskii, 49, 197101 Saint Petersburg, Russia; prithu.roy@fresnel.fr; 3 Higher School of Engineering Physics, Peter the Great Saint Petersburg Polytechnic University, Politekhnicheskaya 29, 195251 Saint Petersburg, Russia; 4 Institute for Analytical Instrumentation RAS, Ivana Chernykh, 31-33, lit. A, 198095 Saint Petersburg, Russia; 5 Center for Photonics and 2D Materials, Moscow Institute of Physics and Technology, 9 Institutskiy Lane, 141701 Dolgoprudny, Russia

**Keywords:** nanowire, nanodisc, GaP, GaPAs, infrared, photonics, emitter, cavity, waveguide

## Abstract

Tailorable synthesis of axially heterostructured epitaxial nanowires (NWs) with a proper choice of materials allows for the fabrication of novel photonic devices, such as a nanoemitter in the resonant cavity. An example of the structure is a GaP nanowire with ternary GaPAs insertions in the form of nano-sized discs studied in this work. With the use of the micro-photoluminescence technique and numerical calculations, we experimentally and theoretically study photoluminescence emission in individual heterostructured NWs. Due to the high refractive index and near-zero absorption through the emission band, the photoluminescence signal tends to couple into the nanowire cavity acting as a Fabry–Perot resonator, while weak radiation propagating perpendicular to the nanowire axis is registered in the vicinity of each nano-sized disc. Thus, within the heterostructured nanowire, both amplitude and spectrally anisotropic photoluminescent signals can be achieved. Numerical modeling of the nanowire with insertions emitting in infrared demonstrates a decay in the emission directivity and simultaneous rise of the emitters coupling with an increase in the wavelength. The emergence of modulated and non-modulated radiation is discussed, and possible nanophotonic applications are considered.

## 1. Introduction

Nowadays, nanophotonic structures play an important role in the development of future information technologies as key elements of integrated optical circuitry [1]. Advanced photonic solutions have a remarkable impact on the semiconductor industry, allowing for the generation, processing and transmission of optical signals at the nanoscale [2]. This field is known to be the most promising in terms of energetic efficiency and an increase in operating frequencies.

Low-dimensional nanostructures, such as quantum dots (QDs), have proven themselves as very efficient light emitters due to both photonic and electronic spatial restrictions [3]. The developed epitaxial growth techniques allow for the synthesis of vertically stacked QD arrays in wide-gap thin-film matrices [4]. Despite the lack of lateral arrangement, these nanoheterostructures have been successfully employed in efficient lasers and other optoelectronic devices [5,6].

Another important example of nanostructures used in the fabrication of nanophotonic elements is semiconductor nanowires (NWs). These nanostructures can be fabricated with the use of conventional epitaxial techniques, e.g., molecular beam epitaxy [7] and chemical vapor deposition [8], and rather technologically feasible techniques, such as hydrothermal synthesis [9]. The advances of NWs for emerging semiconductor technologies compared to their thin-film counterparts include the possibility of growth on lattice-mismatched substrates [10], high crystallinity [11] and peculiar geometry, promising both in terms of optical [12] and electronic properties [13,14,15]. The latter makes NWs especially important for the fabrication of nano-sized conductive elements [16]; sensoric components [17]; and passive and active photonic structures, including waveguides [12], cavities [18] and emitters [19,20].

An important property of NWs is the ability to vary their crystallinity and chemical composition both in vertical (axial heterostructures) and lateral (core–shell heterostructures) directions, thus providing growth possibilities that are unavailable with other structure geometries [21,22]. Within this approach, different elements and devices, such as nanoscale LEDs [23], lasers [24] and solar cells [6], and even more advanced devices, such as single [25,26,27] and entangled photon emitters [28,29] based on semiconductor NWs, were developed. One of the most intriguing possibilities for nanophotonics and optoelectronics relates to the synthesis of vertically stacked nano-sized insertions in NWs and their further passivation with the deposition of the wide-gap shell layer [30]. This provides control over the coupling of the emission and the increase in the quantum efficiency due to the resonant properties of the NW acting as an optical cavity.

Gallium phosphide (GaP) is a mature semiconductor material. In terms of its optical properties, GaP is low loss over almost the entire visible and IR ranges, and it is optically dense, making it priceless for the fabrication of waveguides and cavities [31,32]. Despite being an indirect bandgap material, GaP can be alloyed with other isovalent elements, such as, N, Al, In etc., making it a direct bandgap. This chemical variation provides opportunities for the fabrication of active photonic elements based on GaP NWs.

Several groups previously addressed the optical properties of heterostructured NWs. Many efforts have been put into the optimization of the structural, chemical and geometrical parameters of the nano-sized insertions for precise control over the emission [33,34]. Other groups demonstrated the crucial influence of the structure geometry on light coupling and waveguiding [35,36], provoking the excitation of specific photonic modes and corresponding near-field distributions and unveiling new pathways for device applications. Strongly anisotropic scattering phenomena in semiconductor nanowires were also previously reported [37,38]. Few works reported control over the emission pattern of the NW arrays [39,40], single QD/NW emitters [41,42] and hybrid metal NW structures [43], motivating the development of two-dimensional metasurface lenses and other photonic solutions.

To date, investigations of the optical response in heterostructured NWs mostly considered emission from the nanostructure arrays, its efficiency and far-field patterns governed by the interest in the development of large-scale light-emitting devices. However, considering heterostructured NW as a nanophotonic element, both spectral and spatial characteristics of the generated emission should be analyzed. Here, we report on the study of the micro-photoluminescence (micro-PL) response generated in multiple nano-sized GaPAs insertions in a GaP NW and discuss its anisotropic nature and spectral peculiarities occurring due to the structure geometry. The study is further generalized with the modeling of the insertion emission in IR, which can be obtained with the tailoring of the alloy chemical composition.

## 2. Materials and Methods

### 2.1. Nanowire Synthesis

Axial GaP/GaPAs NW heterostructures were grown via the self-catalyzed vapor–liquid–solid (VLS) mechanism on Si (111) substrates using solid-source molecular beam epitaxy (MBE) Veeco GEN-III system. Si substrates were treated with the Shiraki cleaning procedure [44] and finished with wet chemical oxidation in a base piranha solution (ammonia-peroxide water mixture with a ratio of 1:1:3) [45]. Prior to the NW formation, oxidized Si (111) substrates were thermally annealed in MBE chamber at 760 °C for 30 min in order to promote the formation of pinholes in the surface oxide layer, which serve as Ga catalytic droplet nucleation centers. The detailed procedure and study on the self-catalytic GaP NW formation on Si (111) are presented in [46]. The group-III and -V element fluxes were controlled with Bayard–Alpert vacuum gauge, measuring their beam equivalent pressures. P_2_ and As_4_ beam species were produced by valved cracker cells. As the Bayard–Alpert gauge sensitivity factors differ for P_2_ and As_4_ molecular species, we determined the P/As ratio in terms of stoichiometric group-V flux values found via observation of the transition between group-V and group-III limited growth regimes for GaAs and GaP (001) epilayers.

**Figure 1:**
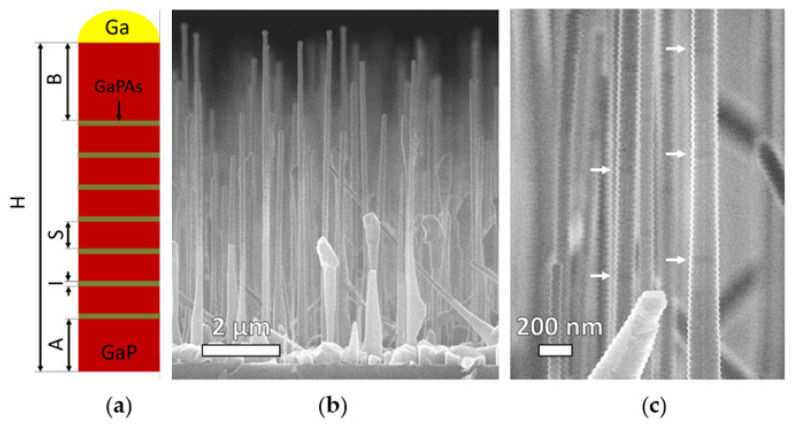
(**a**) Synthesized NW heterostructure schematics (not to scale) with estimated mean dimensions A (stem) = 2 µm, B (top GaP segment) = 2 µm, I (GaPAs insertion) = 50 nm, S (GaP segment) = 600 nm, H (NW length) = 7.8 µm. SEM images of the heterostructured GaPAs/GaP NW array morphology: cross-section view on a cleaved edge (**b**) and close-up NW view with GaPAs NDs marked by white arrows (**c**).

Substrate temperature and Ga cell beam equivalent pressure (BEP) were kept constant during the NW growth and were set at 610 °C and 8 × 10^−8^ Torr (GaP planar growth rate of 3.17 nm/min = 190 nm/h), respectively. Axial NW heterostructure formation was initiated by simultaneous opening of Ga and P_2_ shutters at a P_2_/Ga flux ratio twice the stoichiometric value (V/III BEP ratio set to 12), followed by GaP stem growth with an approximate height of 2 µm for 3000 s with a mean axial growth rate of 0.67 nm/s. Relatively low V/III ratio and growth temperature were chosen to increase the NW diameter to support resonant optical modes in visible spectral range [46]. In situ analysis of the NW crystal structure by the reflection high-energy electron diffraction (RHEED) demonstrated that NWs grow vertically along the Si [111] direction, preserving GaP bulk zinc-blende (ZB) structure. The obtained axial NW heterostructure consisted of 7 identically grown GaPAs nanodiscs (NDs) with an expected thickness of 50 (t = 50 s, mean axial growth rate of 1 nm/s) divided by 6 GaP segments with an expected length of 600 nm (t = 750 s, mean axial growth rate of 0.8 nm/s). NW heterostructure formation was ended by the growth of a GaP segment with a length of 2 µm (for the NW schematics, see Figure 1a).

The composition of GaPAs NDs was targeted to obtain PL emission in the visible-light spectral range according to the procedure described in [47]. The direct bandgap GaP_0.5_As_0.5_ alloy NDs were obtained by tuning the As_4_-to-P_2_ flux ratio (in terms of their stoichiometric values for GaAs and GaP growth) to 2, given the cumulative group-V-to-Ga flux ratio of 3. Here, As/Ga, P/Ga and (As+P)/Ga BEP ratios were set to 24, 6 and 30, respectively. Group-V fluxes were interrupted prior to each ND formation by closing both shutters for 10 s to avoid growth during the adjustment of the As and P needle valves and flux stabilization. During the growth of GaP segment, the arsenic cracking source needle valve and shutter were kept closed. NW formation was interrupted by simultaneously closing both Ga and P_2_ shutters and shutting off the sample heater power, preventing the Ga catalyst droplets from consumption.

### 2.2. Microscopy and Spectroscopy

The as-grown vertical NWs were imaged by means of scanning electron microscopy (SEM, Zeiss Supra 25, Carl Zeiss AG, Oberkochen, Germany). Individual NWs planarized on an auxiliary wafer were characterized with the use of micro-PL technique. The measurements were carried out on LabRAM HR 800 confocal microscope (Horiba Jobin Yvon GmbH, Bensheim, Germany) equipped with a 100× magnification objective (N.A. = 0.9), camera and a stage with piezoelectric controllers for precise positioning of the laser beam and mapping of the optical response. The excitation source was a diode-pumped solid-state 532 nm CW laser (Torus Technology, Telford, England). The optical system focuses the excitation into the Gaussian beam with a diameter of about 1 μm and FWHM of about 300 nm, enabling local excitation of an NW with a high spatial resolution [48]. The optical signal is collected with the same objective.

### 2.3. Modeling

Numerical simulation of the NW optical properties was performed using finite element method (FEM) on commercially available COMSOL Multiphysics software. The simulation was memory intensive due to a large domain size, so the simulation was carried out on a 256 Gb RAM, 10 core server. The adaptive tetrahedral 1 nm (inside the NDs) and 10–100 nm (surrounding of the model) meshes were used to obtain better resolution near the emitters and in the surrounding media. The adaptive meshing reduces the mesh size by one order in comparison to a homogeneous fine mesh, thus speeding up the simulation. Due to the limited capacity of the server, the model considers 4 µm long and 140 nm thick GaP NW with six 50 nm alloyed ND insertions separated by 600 nm GaP segments. The model considers the ITO thin film on the substrate.

## 3. Results

### 3.1. NW Morphology Study and Preparation for PL Characterization

Representative SEM images of the heterostructured GaPAs/GaP NW array morphology are shown in Figure 1b. The resulting epitaxial array demonstrates a mean NW height of 7.8 ± 0.8 µm and an NW diameter of 150 ± 20 nm and 160 ± 55 nm at their top and bottom parts, respectively. It should be noted that on the close-up SEM image presented in Figure 1c, a contrast between the GaP segments and GaPAs NDs can be distinguished, which indicates the formation of axial heterojunctions in the grown NW.

**Figure 2:**
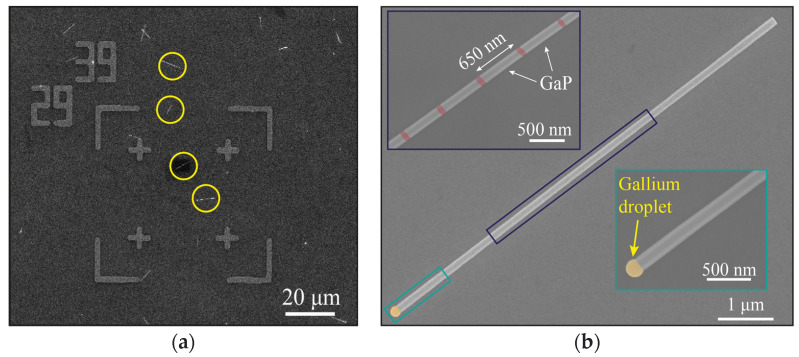
(**a**) SEM image of the marked glass wafer with the drop-casted NWs highlighted with yellow circles, and (**b**) SEM image of individual GaP/GaP_x_As_1−x_ NW heterostructure; the insets show the enlarged views of the segments with GaP_x_As_1−x_ NDs (red colored) and Ga droplet.

For the micro-PL characterization, the NWs were separated from the growth substrate to an auxiliary wafer. To do this, a piece of the as-grown sample was subjected to 1 min sonication in isopropanol (IPA). Then, the NW suspension was dropped on a quartz glass wafer (160 μm thick), providing sufficient optical contrast and allowing for a better coupling and enhancement of the NW cavity Q factor. For the consequent evaluation of the NW longitudinal and lateral dimensions, the glass wafer was prepared for SEM imaging. The preparation process included (1) the covering of the glass substrate with ITO for the facilitated charge drain and (2) the deposition of numbered golden marks (25 nm thick) over a thin Cr adhesion layer with the use of laser lithography and thermal evaporation (Figure 2a).

The fabricated marked substrate allowed for the measurement of the NW dimensions with SEM and the consequent optical characterization of the specific NW with the help of the coordinate grid providing the exact NW location. An SEM image of a single NW is presented in Figure 2b. According to the analysis of the SEM image in Figure 2b, the NW length (8.4 ± 0.05 μm) and diameter (160 ± 10 nm) were evaluated. The gallium droplet (marked in yellow) diameter can be observed to be 190 ± 10 nm. All of the seven GaP_x_As_1−x_ direct gap inserts (marked in red) are contrasted on the SEM images and are found to be 50 nm thick and separated from each other with 600 nm long GaP segments. The first (from the NW bottom) and the last NDs are located 1.9 microns far from the edge and 2.55 microns far from the Ga droplet, respectively. It is important to note that despite the self-catalyzed growth mechanism, where droplets could either inflate or deflate due to a mismatch between the incoming and crystallization Ga species fluxes [46], the studied NW is not tapered; its diameter is stable along the entire length, making the interpretation of the results more straightforward.

### 3.2. Micro-PL Study

**Figure 3:**
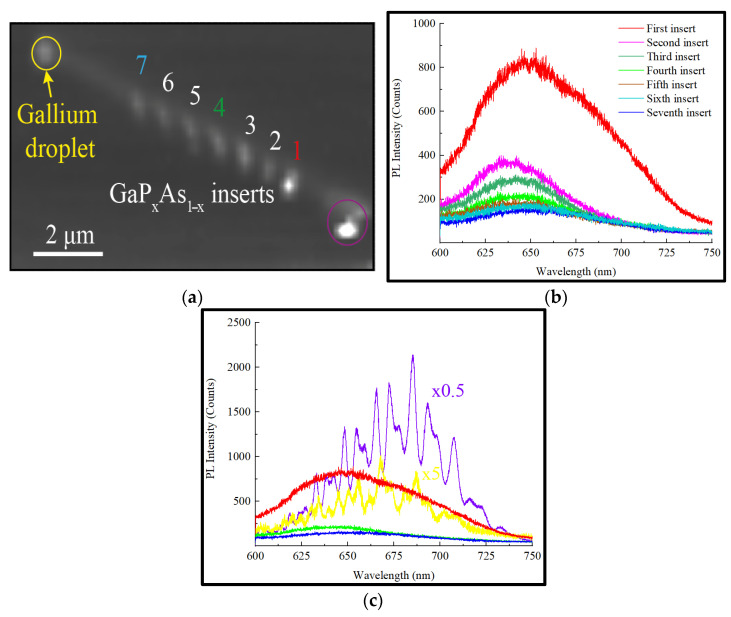
(**a**) RT PL integral intensity map of the studied NW, and (**b**) PL spectra acquired at each of the GaPAs NDs. (**c**) PL spectra measured on the edge side without the Ga droplet (violet, multiplied by 0.5 for visibility); on the edge with the Ga droplet (yellow, multiplied by 5); and at the NDs 1 (red), 4 (green) and 7 (blue).

To study the emission features of the heterostructured NW upon optical pumping in detail, we mapped at room temperature (RT) the PL signal of the NW on the glass substrate imaged in Figure 2b. To provide high resolution of the mapping, the scan step was set at 40 nm in both directions. The obtained gray scale map (Figure 3a) of the PL signal integrated over a 500–800 nm range is in agreement with the NW structure shown in the SEM images. On the map, nine hot spots can be clearly distinguished: two at the edges and the other seven perfectly coinciding with the position of the direct-gap GaPAs inserts. The corresponding spectra at the hot spots were then analyzed. The PL responses corresponding to the NDs are shown in Figure 3b.

The emergence of the PL emission at the ND site means that despite the small NW cross-section and the propagation of the excitation perpendicular to the NW axis, the focused light can excite an individual ND. All of the observed spectra are centered between 640 and 650 nm with an emission band from 600 to 700 nm. The emission centerline corresponds to the arsenic content of about x = 0.6 according to Vegard’s law [49], which is close to the expected P:As ratio of 1. The PL intensity varies from one ND to another, with the most intense signal observed at the disc nearest to the NW edge (first ND).

Figure 3c demonstrates the PL spectra collected in the vicinity of the NW top (yellow curve) and bottom (violet curve) edge facets. Both spectra are found to be strongly modulated, unlike the emission collected in the vicinity of the NDs. The modulation occurs when the Fabry–Perot (F-P) resonance condition is met [50,51]. The NW edges, first, correspond to the field maxima for the standing waves, fulfilling the F-P condition, and, second, act as efficient scattering cites, which is why we observe a high-intensity modulated signal here. However, NDs are weak scatterers due to a low GaP/GaPAs optical contrast and small ND thickness, leading to the absence of the ND response modulation. A sufficient part of the ND radiation is directed into the cavity volume and is modulated, while a smaller part emerges from the side surface unmodulated, according to the obtained spectra in Figure 3b.

Despite having the same spectral structure, the response collected near the Ga droplet is found to be one order less intense compared to the signal at the opposite NW edge (see Figure 3c). This effect is further discussed.

### 3.3. Modeling Results

For a deeper understanding of the observed emission features, we performed numerical modeling. In the experiment, we excited the NDs with a 532 nm laser, followed by the emission centered near 650 nm. To understand this coupled phenomenon, we performed the simulation and untangled excitation and the ND emission.

**Figure 4:**
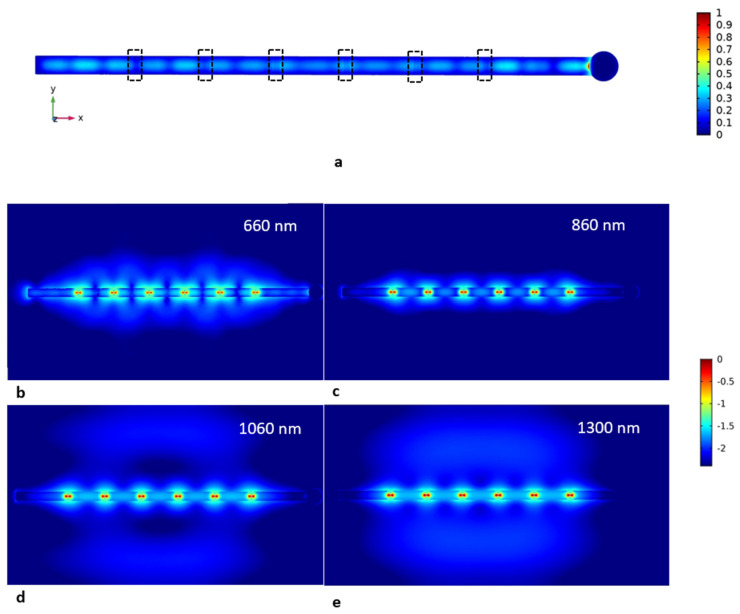
(**a**) Distribution of the electric field in a 4 µm NW with 6 GaPAs insertions (highlighted with a dashed line) excited by 532 nm plane wave travelling along the Z axis and polarized along the X axis. The map shows the field distribution along the center plane of the NW parallel to the substrate surface (XY). The plot is normalized (E/E_0_), where E is the field at a given point and E_0_ is the applied field. Due to the small size of the insertions and low GaP-GaPAs optical contrast, the field distribution is weakly distorted, and NDs are not distinguishable on the map; (**b**–**e**) 4 μm long NW with 6 GaPAs NDs separated by 600 nm GaP segments; each ND is simulated with 50 nm dipole, polarized along the X axis emitting at 650 (**b**), 860 (**c**), 1060 (**d**) and 1300 nm (**e**). The plot is log scale of the normalized field (E/E_0_) to demonstrate faint coupling of the emitters.

Thus, two different models were studied. The first one is the model of the excitation part of the experiment. The idea of this modeling is to show how the field manifests itself in the NW with ND insertions. Therefore, we excited the NW with a 532 nm plane wave directed toward the substrate plane with polarization parallel to the NW main axis. The obtained field pattern is shown in Figure 4a. Two important features are found in the pattern. The image shows that the field is not homogeneous but modulated along the wire due to the occurrence of Fabry–Perot resonances in the NW, which are more prominent near the edges. The second feature is the field localization on the droplet edge due to the reflection, as well as plasmon generation. Since the polarization of the field is parallel to the wire axis, i.e., perpendicular to the metal–dielectric interface, we observe the generation of a localized surface plasmon. This feature in the droplet–NW system is discussed in our previous work [43].

The second simulation is the emission model. In this model, we studied the emission from six insertions of 50 nm thick NDs. This was carried out by exciting the line dipole (50 nm) inside the insertion and observing the field pattern for a 650 nm wavelength corresponding to the GaPAs ND emission band centerline observed experimentally. The calculated field pattern is presented in Figure 4b. The dipoles are orientated along the X axis, and the emission pattern is expected in the Y direction. However, due to the high refractive index in comparison to the surroundings, the NW directs this emission toward the NW edges.

As was discussed in the Introduction, the VLS growth mechanism provides extensive opportunities for bandgap engineering. As such, the use of In allowed us to increase the emission wavelength up to the IR region via the introduction of InGaP and InGaAs solutions. Moreover, additional control of the near-field emission can be obtained with the use of Ga(Al)As alloys. To provide insight into the perspectives of the heterostructured NWs for IR on-chip circuits, we numerically simulated the near-field distribution with insertions emitting at 860, 1060 and 1300 nm. The corresponding maps are presented in Figure 4c–e. The model geometry and dipole orientation are similar to those of the model above.

## 4. Discussion

The obtained experimental results together with the numerical modeling demonstrate that an axially heterostructured NW under an optical excitation behaves as a light source with peculiar spectral and spatial characteristics. Here, we discuss the observed features.

According to the experimental results depicted in Figure 3a,b, the PL intensity is non-uniform throughout the NDs despite their similar geometry. This effect can be explained by two factors. The first factor is the size of the laser spot (~1 μm), which can lead to partial coupling of excitation at the edge facet when the laser spot is centered at the ND placed in the vicinity of the edge, followed by subsequent more efficient ND excitation. However, the results of the excitation modeling presented in Figure 4a demonstrate the non-uniform field pattern inside the NW governed by its resonant property. Thus, the location of the ND affects the coupling of the excitation, which can undergo either constructive or destructive interference, specific to the ND location and the size and shape of the NW. The difference in the NDs’ emission spectra is unlikely related to their chemical composition variation. The NW is not tapered, meaning preservation of the catalyst particle size during growth, which leads to uniformity of the efficient growth fluxes, so the disc’s chemical composition is expected to be similar. Due to the axial polarization of the dipoles (corresponding to the polarization of the excitation), the dipole–dipole coupling of emitters can be enhanced by the choice of the distance between them.

According to Figure 3c, the PL response collected at the edge without the Ga droplet turned out to be sufficiently higher than the emission at the droplet site. The demonstrated phenomenon can be the subject of the absorption of the PL signal by the plasmonic droplet. This effect, however, should lead to the decay of the resonant properties providing a weak F-P modulation of the PL signal. Moreover, as was demonstrated previously [43], the Ga droplet acts more as an antenna guiding the wave into the NW rather than an absorber. The simulation results presented in Figure 4 demonstrate that the Ga nanoparticle enhances the reflective behavior of the facet with the corresponding increase in the field collected at the opposite edge. Moreover, it seems that the Ga droplet is an inefficient scatterer, while the intensity of the outcoupled emission is demonstrated to be higher at the edge without the droplet according to Figure 4b. The modeling also demonstrates the effect of the edge on the emission coupling—the closer the ND to the edge, the more efficient the coupling in comparison to that in the center.

**Figure 5:**
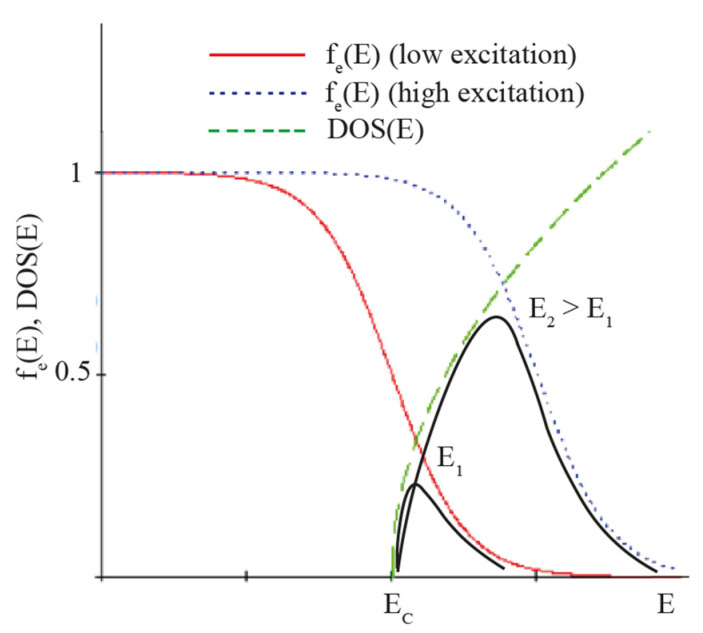
DOS and electron Fermi–Dirac functions for different excitation powers and their overlap (black curve) in both cases.

A comparison of the PL spectra in Figure 3 demonstrates a spectral shift of about 20 nm between the PL peak positions of the emission collected at the NW edge and NDs, with the first one displaying a red shift. To explain this phenomenon, it is necessary to consider that the PL intensity is proportional to the concentration of the charge carriers. The optically pumped semiconductor is in a strong nonequilibrium; therefore, the concept of quasi-Fermi levels can be applied. Let us consider the concentration of electrons. By definition, this concentration (n_e_) in the conduction band can be calculated with the following expression:

(1) $$n_{e}=\overset{+\infty }{\underset{E_{C}}{\int }}\mathrm{DOS}\left(E-E_{C}\right)f_{e}\left(E\right)\mathrm{dE}$$

where E_C_—conduction band bottom level, and f_e_(E)—Fermi–Dirac function depending on the quasi-Fermi level. Therefore, the electron concentration is, in fact, the area under the curve, which is the product of the density of states (DOS) and the Fermi–Dirac distribution function containing the quasi-Fermi level for electrons. In our experiment, the excitation and collection are with the same objective, and when the laser beam is directed toward an ND, the excitation in the ND is much higher compared to the case where it is located at the NW edge. Consequently, in the first case, the nonequilibrium is stronger and the quasi-Fermi level of electrons is higher than that in the second case. As such, the Fermi–Dirac distribution function shifts toward the higher energies, and the peak of its overlap with the DOS also shifts toward the higher energies (see Figure 5). The position of this peak and the entire curve of the overlap function govern the PL spectrum of the interband transitions. As a result, a weaker excitation occurs when the beam is focused at the NW edge, leading to its red shift.

Figure 4c–e show the near-field pattern for the insertions emitting in near IR. GaP is a low-loss material with the near-zero imaginary part of the refractive index. However, for the provided wavelengths, the real part of the refractive index decreases from 3.4 (650 nm) to 3.1 (1300 nm) [52]. This affects the directivity of the NW: as the wavelength increases, the light coupling in the NW reduces with the corresponding fall of the emission directed toward the edges. Thus, both the outcoupling by the left edge and the reflection by the other decrease. This effect is clearly seen with the diffused light in the surrounding medium, where intensity rises with the increasing wavelengths.

However, the coupling efficiency of the NDs increases with the wavelength, followed by the densification of the field intensity between the NDs inside the NW. This feature can enhance the synergy of the collective resonant emission at even longer wavelengths. Even though the directivity decays with the wavelength, the observed effect corresponds to the loss of the waveguiding property. Thus, adjustment of the NW diameter can be used to tailor the emission pattern, paving the way to applications such as broad-band waveguide resonators.

The results of the study demonstrate that the heterostructured GaP/GaPAs NW acts as a cavity with emitters in the form of ND, representing a system with spectrally and spatially non-uniform emission upon optical pumping and characterized by the following:

- The emission of an individual ND is anisotropic and is coupled inside the NW, while part of it is emitted outside the NW;
- The emission outside the NW in the vicinity of an ND is due to the PL of the ND and not due to the scattering of the light propagating in the cavity, so this emission should be governed by the bandgap of the ND and can be controlled via the variation of its chemical composition;
- The non-uniform distribution of the excitation light along the NW governed by the wavelength and NW geometry affects the PL intensity of each emitter;
- Outcoupling is the most efficient at the NW edge without the catalyst droplet;
- The emission outcoupled at the NW edge is modulated due to the NW geometry promoting its resonant optical properties.

**Figure 6:**
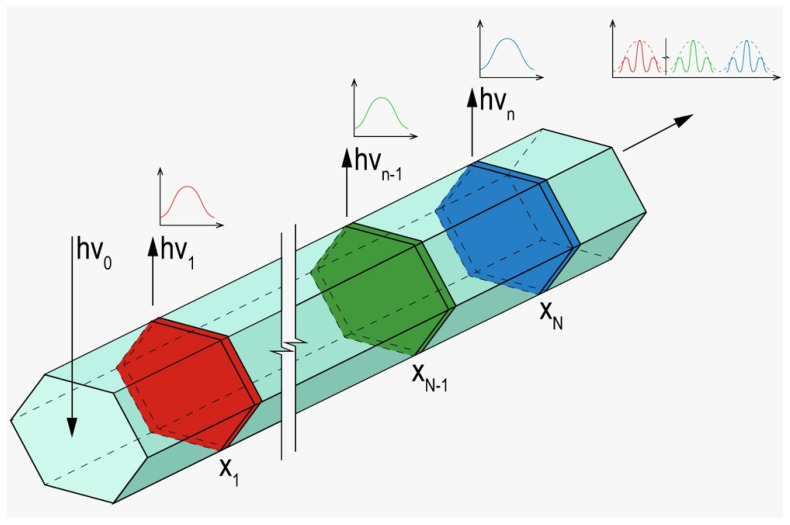
Schematics of the heterostructured NW with multiple NDs characterized by the corresponding PL emission with a characteristic photon energy hν_k_. hν_0_—excitation.

An artistic impression of the discussed phenomena is presented in Figure 6.

The obtained results shed light on new pathways for advanced photonic applications. For example, the spectrum of the PL emission outcoupled at the edge of an NW, schematically shown in Figure 6, can be controlled via the variation of the excitation wavelength. To obtain the spectrally variable emitter based on this approach, NDs with different bandgaps should be precisely positioned inside an NW so that the shift in the excitation wavelength leads to a spatial shift of the NW mode maxima, followed by the switch of an excited emitter. That is, by changing the input signal, photoluminescence can be initiated in a specific ND. Modulation of the PL response due to the geometry of an NW is considered to be another key within the approach to controlling the emission spectrum. We should also note that the near field in the vicinity of each ND is affected by the change in the excitation wavelength. The discussed effects can be used, for example, in the development of optically controlled variable emitters and data processing systems. Utilization of the plasmonic effects provided by a Ga catalyst particle can also widen the possibilities for the implementation of heterostructured NWs in photonic devices [53,54]. A proper optical scheme will allow for the use of the discussed nanostructure as an element of a logic circuit or an analog-to-digital photonic converter.

## 5. Conclusions

To conclude, in this work, we experimentally and theoretically study the spectral and spatial features of the PL excited in the GaPAs insertions in a GaP nanowire. The results demonstrate several interesting phenomena governed by the geometry of the studied system. The NDs demonstrate anisotropic emission, which can be outcoupled both in the vicinity of the ND and the NW edge. Due to the resonant properties of the NW, the emission outcoupled at the NW edge exhibits strong modulation, while the emission at the ND site is found to be unmodulated and less intense. Another interesting feature of the system is the role of the Ga droplet, which directs the emission inside the NW toward the opposite edge. Additional modeling of the NW with IR emitters demonstrates the effects of the field pattern change leading to the weaker directivity of the emission along the NW axis and a more efficient coupling of the emitters, providing several opportunities for the resonant emission with multiple insertions or quantum dots in an NW cavity. The investigated results are discussed in terms of possible applications in nanophotonics.

## Data Availability

The data presented in this study are available on request from the corresponding author.
